# Structure and IR Spectroscopic Properties of HNCO Complexes with SO_2_ Isolated in Solid Argon

**DOI:** 10.3390/molecules26216441

**Published:** 2021-10-25

**Authors:** Justyna Krupa, Maria Wierzejewska, Jan Lundell

**Affiliations:** 1 Faculty of Chemistry, University of Wroclaw, Joliot-Curie 14, 50-383 Wroclaw, Poland; maria.wierzejewska@chem.uni.wroc.pl; 2 Department of Chemistry, University of Jyväskylä, P.O. Box 35, FI-40014 Jyväskylä, Finland

**Keywords:** hydrogen bond, van der Waals interaction, Matrix isolation, vibrational spectroscopy, computational chemistry

## Abstract

FTIR spectroscopy was combined with the matrix isolation technique and quantum chemical calculations with the aim of studying complexes of isocyanic acid with sulfur dioxide. The structures of the HNCO⋯SO_2_ complexes of 1:1, 1:2 and 2:1 stoichiometry were optimized at the MP2, B3LYPD3, B2PLYPD3 levels of theory with the 6-311++G(3df,3pd) basis set. Five stable 1:1 HNCO⋯SO_2_ complexes were found. Three of them contain a weak N-H⋯O hydrogen bond, whereas two other structures are stabilized by van der Waals interactions. The analysis of the HNCO/SO_2_/Ar spectra after deposition indicates that mostly the 1:1 hydrogen-bonded complexes are present in argon matrices, with a small amount of the van der Waals structures. Upon annealing, complexes of the 1:2 stoichiometry were detected, as well.

## 1. Introduction

Isocyanic acid (HNCO) is an intriguing molecule that has been widely studied both experimentally and theoretically [1,2,3,4,5,6,7,8,9,10,11,12,13,14,15,16,17,18,19]. It is one of the simplest molecules containing all four important biogenic elements (C, N, O, and H). There are four open-chain isomers identified for CHNO species: fulminic acid (HCNO), isofulminic acid (HONC), cyanic acid (HOCN) and isocyanic acid (HNCO), with the latter being the most stable. Shapley and Bacskay [18] have shown by various theoretical methods that in addition to these open-chain structures, several cyclic and branched HNCO isomers of much higher energies can exist. Experimentally, structure of HCNO isomers was determined by microwave and infrared spectroscopies. Bondybey et al. [6] characterized HNCO and HCNO molecules isolated in low temperature matrices, as well as their photoproducts upon UV photolysis. Infrared spectra of all four HCNO isomers and their isotopomers isolated in argon matrices were reported Teles et al. [8]. Furthermore, Pettersson et al. [12,13] used infrared and LIF spectroscopies as detection methods to follow the UV photolysis of HNCO isolated in xenon matrices, and identified plethora of interesting photoproducts. Among them were H_2_NCO radical, HCO, HXeNCO, HXeCN, HXeNC, and HXeH. It was also found that irradiation of formamide in solid Xe at 193 nm led to HNCO+H_2_ binary system as a product specific for the xenon matrix [20]. Some papers on properties of less stable isomers of HNCO have been published as well [21,22].

Isocyanic acid can be found in urban environments and biomass-burning-affected regions [23]. It plays an important role in combustion reactions as for instance RapreNOx processes in which HNCO reduces NO and other toxic nitrogen oxides via a complex chain reaction [24,25,26,27]. HNCO was also detected in 1973 in the interstellar medium [28], and has been considered in several astrophysical surrounds as well [29].

Although the properties of HNCO and its isomers are now relatively well recognized, only a small number of HNCO complexes have been studied so far. Raunier et al. [30] investigated thermal reactivity of HNCO with water ice and presented also infrared spectra of the 1:1 HNCO complex with H_2_O isolated in an argon matrix. The complex was found to present shifts of the νNH and ν_as_NCO vibrational modes to be −215 and +13 cm^−1^, respectively, relative to the HNCO monomer modes. Based on the data, it was concluded, in agreement with the performed MP2/6-31G(d,p) calculations, that HNCO interacts with H_2_O via NH⋯O hydrogen bond. Sałdyka and Mielke [31] studied photodecomposition of formohydroxamic acid isolated in solid argon and detected formation of two different structures of the 1:1 HNCO complex with water. One of them corresponds to the structure reported by Raunier et al. [30], in which the NH group of isocyanic acid acts as a proton donor toward the oxygen atom of water molecule. For the second structure identified the water molecule acts as a proton donor toward the nitrogen atom of HNCO. Similarly, photodecomposition of N-hydroxyurea and acetohydroxamic acid in argon matrices led to the formation of HNCO complexes with NH_2_OH and CH_3_OH, respectively [32,33]. Very recently, a report on the photolysis of 1,2,5- and 1,3,4-oxadiazoles was published [34]. Here, laser photolysis of 1,3,4-oxadiazole at 220 nm generated HCN⋯HNCO and HCN⋯HOCN complexes, whereas upon a secondary photolysis by a hydrogen lamp, three isocyanide complexes HCNO⋯HNC, HNCO⋯HNC and HNC⋯HOCN were detected and characterized.

Sulfur dioxide is also a very important atmospheric component. It is emitted to the atmosphere from a variety of sources [35,36]. Weak molecular complexes containing SO_2_ have received considerable attention since they are considered to affect the mechanism of its oxidation [37,38,39,40,41,42]. Due to the negative impact of sulfur dioxide on human health, various methods have been developed for its capture [43].

In this paper, we present results of our studies of complexes formed between isocyanic acid (HNCO) and sulfur dioxide. The study relies on low-temperature matrix isolation infrared spectroscopy (MI-FTIR) and computational chemistry as the tools of investigation. MI-FTIR is a successful technique for studying both van der Waals and hydrogen bonded complexes, providing valuable information on the studied molecular systems. One of the most prominent scientists in the field of matrix isolation infrared spectroscopy was Dr. Austin Barnes, who made a great contribution to the development of this method and enriched the subject with many interesting publications and reviews [44,45,46,47,48,49,50,51,52,53,54]. Dr. Barnes used the MI-FTIR method to unravel the secrets of many molecules and their complexes, and here we follow in his footsteps to gain experimental and computational insight into a molecular complex that could have atmospheric or astrochemical interest.

## 2. Results and Discussion

### 2.1. Computational Results

#### 2.1.1. Structure and Energetics of the 1:1 HNCO⋯SO_2_ Complexes

**Figure 1:**
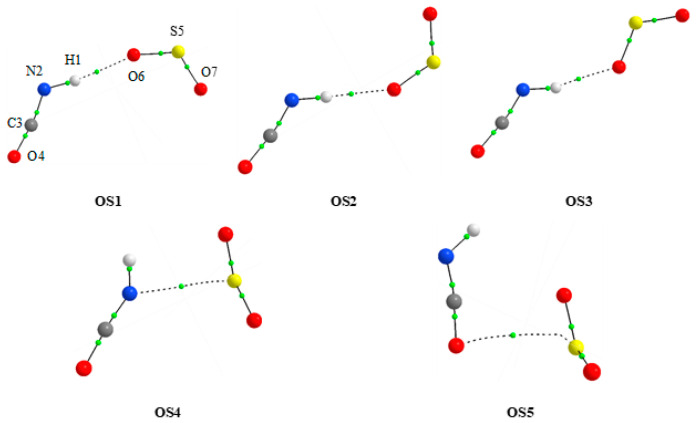
The MP2 optimized structures of the 1:1 complexes of HNCO with SO_2_.

At all levels of theory, five minima were found for the 1:1 HNCO⋯SO_2_ complex. Figure 1 shows the MP2 optimized geometries of these molecular complexes. Their MP2 calculated values of intermolecular distances and angles are presented in Table 1. Cartesian coordinates of the optimized species are provided in Table S1 in Supplementary Material. The values of two important topological AIM [55] parameters: the electron density ρ(r) and its Laplacian ∇^2^ρ(r) at the critical points are also shown in Table 1. Additionally, the positions of the bond (3,-1) critical points derived from AIM calculations are depicted in Figure 1. These data provide valuable information on different kinds of non-covalent bonds and intermolecular interactions in the studied complexes.

Out of five stable 1:1 species, three structures (OS1, OS2 and OS3) are characterized, as supported by AIM parameters, by the presence of a weak N-H...O hydrogen bond. These complexes are planar and differ by the arrangement of the SO_2_ moiety with respect to the HNCO molecule. Two other 1:1 complexes are of the van der Waals (vdW) type. The S⋯N and S⋯O contacts were found for the OS4 and OS5 species, respectively. It is worth mentioning that the attempts undertaken to optimize the structure in which the N-H group interacts with the sulfur atom of the sulfur dioxide were unsuccessful.

Table 2 presents energetic parameters together with the abundance and dipole moment values obtained for HNCO⋯SO_2_ complexes (1:1) using MP2, B3LYPD3 and B2PLYPD3 methods. These data reveal that the order of the interaction energies in the case of B3LYPD3 calculations differs slightly from those obtained by the MP2 and B2PLYPD3 methods. The same is true for the stability order (ΔE). All three hydrogen bonded complexes are, according to MP2 and B2PLYPD3, more stable than those containing vdW bonds, whereas at B3LYPD3, two structures with hydrogen bonds (OS1, OS2) and one with the vdW interaction (OS5) are the three most stable species. In general, the interaction energies calculated for all five 1:1 complexes are similar in magnitude, and fall within the range of 11.1–12.7, 13.5–14.9 and 12.3–13.8 kJ mol^−1^, for the MP2, B3LYPD3 and B2PLYPD3 methods, respectively. It is worth noting that the relative Gibbs free energy values favor, at all three levels of theory, the hydrogen-bonded species and, in consequence, their estimated gas phase abundance is much higher than those of the van der Waals complexes. It is also worth noting that the optimized structures of the HNCO⋯SO_2_ complex with three hydrogen-bonded and two van der Waals species are very similar to those reported for HNCS⋯SO_2_ interaction [38].

#### 2.1.2. Structure and Energetics of the 1:2 and 2:1 HNCO⋯SO_2_ Complexes

**Figure 2:**
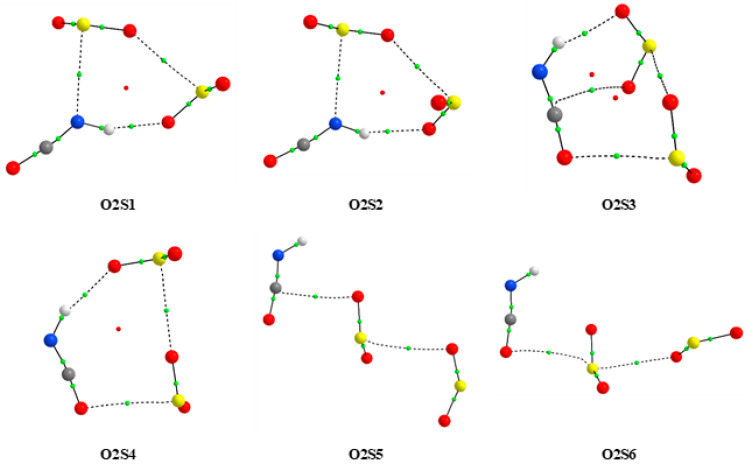
The MP2 optimized structures of the 1:2 complexes of HNCO with SO_2_.

According to the calculation methods used, six and ten minima are present for 1:2 and 2:1 complexes, respectively. Figure 2 shows the optimized geometries of 1:2 complexes and those of the 2:1 composition are presented in Figure S1 in the Supplementary Materials. Cartesian coordinates of all optimized species are gathered in Table S1. The analogous information as for 1:1 species (Table 1) derived from AIM calculations for 1:2 and 2:1 complexes is presented in Tables S2 and S3 in Supplementary Material.

The analysis of the optimized structures of the HNCO complexes with SO_2_ of the 1:2 stoichiometry (see Figure 2) reveals that, similarly to the 1:1 species, the N-H⋯O hydrogen bond and different types of van der Waals interaction contribute to the stability of these complexes. The obtained values of the AIM parameters presented in Table S2 confirmed existence of such non-covalent interactions between particular moieties of the aggregates. Table 3 gathers their interaction energy values which fall into the range of 20.5–34.1 (MP2), 26.2–41.7 (B3LYPD3) and 23.9–38.8 kJ mol^−1^ (B2PLYPD3). Both types of interactions within the aggregates were found for the 2:1 complexes; however, a higher contribution of the NH...N and NH⋯O hydrogen bonds was observed. Slightly higher interaction energy values for these forms were estimated in the range of 22.6–41.0 (MP2), 29.2–47.2 (B3LYPD3) and 25.9–44.1 kJ mol^−1^ (B2PLYPD3). The results obtained for these 2:1 species are presented in Figure S1 and Tables S3 and S4 in the Supplementary Materials.

### 2.2. Matrix Isolation Infrared Spectra

First, separate experiments were conducted for HNCO/Ar and SO_2_/Ar matrices, and the spectra of monomeric species obtained agreed with those published in the literature, [8,56,57,58]. As explained by Cugley and Pullin [58] and Teles et al. [8], the νNH stretching region (ν_1_) of HNCO is characterized by three bands of the split rotational 0←1 transition and two components of the absorption due to the 0←0 transition which originate from Fermi resonance. The values of the unperturbed vNH fundamental were estimated, on the basis of the positions of the Fermi resonance doublet, at 3511.3 cm^−1^ [8]. In turn, for the SO_2_ monomer isolated in argon matrices doublets were observed in each fundamental vibrational mode region. Upon annealing, the intensity of the low wavenumber component of the doublets decreased whereas the high wavenumber band was nearly temperature insensitive. Such a behavior arises from two types of site at which SO_2_ molecules reside: stable sites (cubic close packing) and metastable sites (hexagonal close packing) [56].

Of the six infrared active vibrations of HNCO, two ν_s_NCO stretching and γNCO deformation are very low in intensity. Two other vibrations, namely δNH and δNCO are strongly coupled and their behavior upon complexation is expected to be ambiguous and difficult to follow. The two remaining bands of νNH and ν_as_NCO stretching modes are characterized by relatively high intensity, and they should be good markers for providing information about the structure of the studied complexes.

#### 2.2.1. HNCO⋯SO_2_ Complexes of the 1:1 Stoichiometry

When both HNCO/Ar and SO_2_/Ar gas mixtures were co-deposited at 15 K (10 K for measurements), several new bands appeared when compared to the spectra of the parent molecules in solid argon. Table 4 summarizes the selected wavenumber shifts calculated for the 1:1 complexes using the three computational methods compared to the experimental results. In addition, theoretical infrared wavenumbers and intensities obtained for the monomers and the 1:1 complexes are presented in Table S5 in the Supplementary Material. It is known that the intermolecular vibrational modes of weak hydrogen bonded and van der Waals complexes are strongly anharmonic [52,59,60]. Therefore, for the experimental data analysis and discussion, the wavenumber shifts were mostly used.

**Figure 3:**
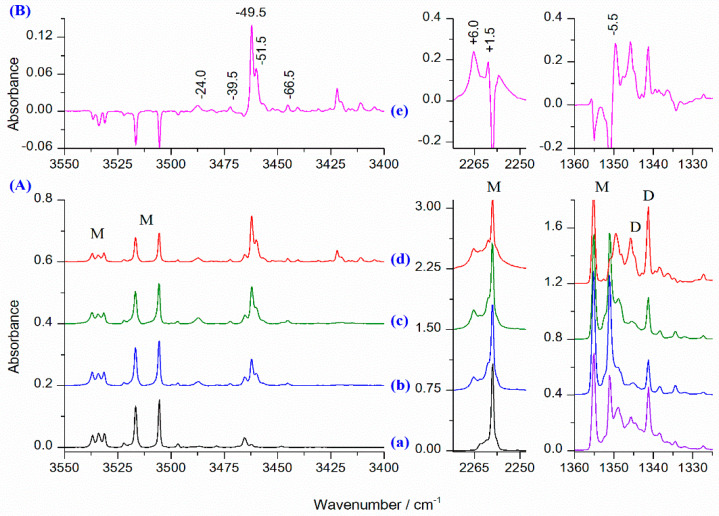
(**A**) The νNH, ν_as_NCO and ν_as_SO_2_ regions in the spectra of matrices: HNCO/Ar = 1/6000 or SO_2_/Ar = 1/1400 (after 5 min at 30 K/10 K) (**a**), HNCO/SO_2_/Ar = 1/2/5600 (**b**), HNCO/SO_2_/Ar = 1/4/5600 (**c**), and matrix (**c**) after 10 min at 30 K/10 K (**d**). (**B**) The difference spectrum (**e**) obtained by subtracting the spectrum (**a**) from the spectrum (**d**) (pink trace). The letters M and D denote HNCO and SO_2_ monomers and dimer bands, respectively.

Figure 3 shows the νNH, ν_as_NCO and ν_as_SO_2_ stretching vibration regions of the spectra of the HNCO/SO_2_/Ar matrices at two different HNCO/SO_2_ ratios obtained directly after deposition and one of them obtained upon annealing at 30 K/10 K. The corresponding fragments of the HNCO/Ar and SO_2_/Ar spectra are also shown for comparison purposes. The respective ranges of the difference spectrum are presented at the top of Figure 3 showing changes upon complexation and annealing the matrix at 30 K/10 K. Directly after deposition of the HNCO/SO_2_/Ar mixtures (traces b and c in Figure 3), a new, relatively intense band at 3462.0 cm^−1^ with a shoulder at 3460.0 cm^−1^ appeared in the νNH stretching mode region, as well as three much weaker bands at 3487.5, 3472.0 and 3445.0 cm^−1^. The intensity ratio of all these bands is the same at both concentrations applied, indicating that they originate from the 1:1 HNCO⋯SO_2_ complexes. These bands are accompanied by absorptions in the ν_as_NCO stretching region at 2265.0 and 2260.5 cm^−1^.

According to the performed calculations, there should be two other modes of relatively high intensity for 1:1 complexes, namely asymmetric stretching ν_as_SO_2_ and in-plane deformation δNH (coupled with δNCO bending). As shown in Figure 3, a new band appeared upon complexation in the ν_as_SO_2_ region at 1349.5 cm^−1^ with the red shift of 5.5 cm^−1^ compared to the ν_as_SO_2_ band of the sulfur dioxide monomer isolated in a stable site. This absorption is hardly seen upon deposition because it is close to one of those assigned to the SO_2_ dimer [57]. However, it is apparent upon annealing at 30 K. Our calculations predicted a red shift in the range 1 and 8 cm^−1^ for all five 1:1 HNCO⋯SO_2_ complexes. Therefore, the ν_as_SO_2_ mode may not be considered to be a good indicator of the structure of the studied species.

In turn, as it comes to the δNH deformation mode, it has been suggested to give rise to a very broad absorption and was not observed in the spectra of HNCS⋯SO_2_ in solid argon [38]. It is probably that a similar situation is true for the HNCO⋯SO_2_ complex, since the δNH mode could not be localized in the present spectra.

Structure determination of the complexes formed between HNCO and SO_2_ seems to be a difficult task. As can be seen from Table 2, the differences between the interaction energies (E_int_) of the five 1:1 HNCO⋯SO_2_ complexes are less than 1.55 kJ mol^−1^ and the same is true for the relative energy ΔE. Larger differences are predicted for the relative Gibbs free energies. For the hydrogen bonded species, the differences are less than 2 kJ mol^−1^ whereas for the OS4 and OS5 complexes the ΔG values are, depending on the method used, between 4.45 and 5.60 kJ mol^−1^. These values determine the population which are clearly higher for the OS1, OS2 and OS3 complexes compared to the OS4 and OS5. Therefore, it can be expected, assuming that the gas equilibrium is frozen in the low temperature matrices upon deposition, that the hydrogen bonded systems will predominate in the studied matrices.

As is evident from Table 4, where the calculated wavenumber shifts for the key modes for different 1:1 HNCO⋯SO_2_ complexes are gathered, none of the three computational methods gives fully satisfactory agreement between theoretical and experimental values. However, taking into account both the presented spectroscopic data and the population estimated for the 1:1 complexes, tentative conclusions can be drawn regarding their structure in the studied argon matrices. Thus, the most intense band of the HNCO⋯SO_2_ complex at 3462.0 cm^−1^ (Δν = −49.5 cm^−1^) with a shoulder at 3460.0 cm^−1^ (Δν = −51.5 cm^−1^), as well as a weak band at 3445.0 cm^−1^ (Δν = −66.5 cm^−1^), may be assigned to the νNH mode in the hydrogen bonded structures (OS1–OS3). The calculated red shift for this vibration is overestimated, even though values of two other shifts presented in Table 4 fit relatively well to the experimental values. In turn, two weak absorptions at 3472.0 and 3487.5 cm^−1^ characterized with smaller νNH shifts (Δν = −39.5 and −24.0 cm^−1^, respectively) may originate from the van der Waals structures (OS4-OS5). The intensity of all these bands increased in a similar way when the matrix was annealed at 30 K. 

The νNH bands are accompanied by two new bands in the ν_as_NCO region at 2260.5 and 2265.0 cm^−1^ characterized by similar behavior upon annealing. These bands are blue shifted by 1.5 and 6.0 cm^−1^ as compared with the HNCO monomer band and the direction of these changes agrees well with those predicted theoretically for the hydrogen bonded HNCO⋯SO_2_ (1:1) complexes however the latter shift is slightly higher than those predicted to fall in the range of 0–2 cm^−1^ (see Table 4). 

#### 2.2.2. HNCO Complexes with SO_2_ of the 1:2 and 2:1 Stoichiometry

As follows from the computational data shown in Table 3, the four 1:2 complexes containing the N-H⋯O hydrogen bond and van der Waals interactions (O2S1–O2S4) are generally more stable than two remaining species with exclusively van der Waals contacts. The O2S1–O2S4 structures are characterized by very similar values of the interaction energy with a maximum difference of 2.26 kJ mol^−1^. Their relative energies are also quite close to each other.

When HNCO/SO_2_/Ar matrices were subjected to 10 min annealing at 30 K, bands of the monomeric HNCO in the resultant spectra (taken at 10 K) decreased, absorptions assigned to the 1:1 complexes increased slightly, and new bands situated at 3440.0, 3422.0 with the 3420.0 shoulder, 3411.0 and 3404.0 cm^−1^ appeared (Figure 3). These absorptions are assigned to the HNCO complexes with SO_2_ of the 1:2 stoichiometry. The wavenumber red shifts of these bands relative to the monomeric νNH are equal to 71.5, 89.5/91.5, 100.5 and 107.5 cm^−1^, respectively. Table 5 presents selected shifts calculated for the six 1:2 complexes. In addition, computed infrared wavenumbers and intensities obtained for the 1:2 complexes (not observed in the present study) are presented in Table S6 in Supplementary material.

Similar to the 1:1 complexes case, none of the three computational methods used reproduces the experimental shift values very well. However, the values presented in Table 5 clearly point to the presence of such 1:2 structures which contain HNCO unit hydrogen bonded to the oxygen atom of one the SO_2_ moiety. Two such structures, i.e., O2S3 and O2S4, show νNH to be red shifted, and the values are close to the experimental values (74 and 82, 68 and 91, 74 and 88 cm^−1^ at MP2, B3LYPD3, B2PLYPD3, respectively). These forms are probably present in the studied matrices, but the two complexes exhibiting larger shifts, O2S1 and O2S2, cannot fully be excluded based on the data available here. The vibrational shifts of the ν_as_NCO mode predicted for the hydrogen bonded 1:2 species are, with one exception, small negative values in the 2–4 cm^−1^ range and are not observed experimentally.

Theoretical infrared wavenumbers and intensities obtained for the HNCO complexes with SO_2_ of the 2:1 stoichiometry are presented in Table S7 in the Supplementary Material. At the experimental conditions applied in these studies bands originating from such species were not identified.

## 3. Computational and Experimental Details

### 3.1. Computational Methods 

To support the analysis of the experimental data, computational studies for the 1:1, 1:2 and 2:1 complexes formed between HNCO and SO_2_ were carried out using the Gaussian16 program package [61]. The initial geometry of the 1:1complexes was based on that found for the HNCS⋯SO_2_ structures [38]. The 1:2 and 2:1 complexes were obtained from the geometry optimized for the 1:1 HNCO⋯SO_2_ species by adding the HNCO or SO_2_ subunit. Structures of the complexes were optimized at the MP2, [62,63,64,65] B3LYPD3 [66,67,68,69,70] and B2PLYPD3 [71,72,73] levels of theory using the 6–311++G(3df,3pd) [74,75] basis set. Optimization of the complexes was performed with the Boys-Bernardi full counterpoise method by Dannenberg [76,77]. The interaction energies were estimated by subtracting the energies of the isolated monomers with the frozen geometry from the energy of the 1:1, 1:2 or 2:1 complexes. The relative energy ΔE and relative Gibbs free energy ΔG for a given structure were obtained by subtracting the E or G values of the most stable complex from the values calculated for the given species. The relative abundance of the complexes was estimated based on the calculated Gibbs free energy values using equation: ΔG = −RT lnK, where ΔG is the difference between Gibbs free energy for two given isomeric forms (at T = 298 K) and K is the equilibrium constant for these species. 

The topological analysis of the electron density (AIM) [55] was performed at the MP2/6-311++G(3df,3pd) level using AIM studio program (Version 19.10.12, Professional [78]). Generally, the applied methods predicted very much similar geometries for the complexes. There are no significant differences in the values of the bond distances and bond angles computed at various levels. The values of dihedral angles were also fairly independent of the method used.

The harmonic vibrational wavenumbers and infrared intensities were calculated at MP2, B3LYPD3 and B2PLYPD3 levels for all optimized complexes in order to support the analysis of the experimentally obtained vibrational spectra. Spectral shifts upon complexation were obtained as the difference between the complex and monomer vibrational wavenumbers.

### 3.2. Matrix Isolation FTIR Studies 

Isocyanic acid was obtained by strongly heating cyanuric acid powder in an evacuated quartz vessel. Generated HNCO vapor passed several times through P_2_O_5_ to remove water and HCN and was condensed in a liquid-nitrogen trap and stored in a 250 mL glass bulb. The gaseous mixtures were prepared by mixing of HNCO and SO_2_ with argon (Messer, 5.0) in two containers in a vacuum system. Matrices were deposited through two jets containing mixtures of molecular subunits with argon onto a CsI window kept at 15 K. Pressure of the gas mixtures and the deposition rates were controlled by piezotransducers (model 902B, MKS Instruments) installed in both deposition lines. Low temperature was attained using a closed cycle helium refrigerator (APD-Cryogenics) and measured directly at the sample holder by a silicon diode sensor coupled with the digital controller (Scientific Instruments). Infrared spectra were taken at 10 K in a transmission mode with 0.5 cm^−1^ resolution by means of a Bruker IFS 66 Fourier Transform spectrometer equipped with a liquid cooled MCT detector.

## 4. Conclusions

For the first time, theoretical and matrix isolation FTIR studies of molecular complexes formed between isocyanic acid and sulfur dioxide are reported. All three computational methods used (MP2, B3LYPD3 and B2PLYPD3) revealed five stable 1:1 complexes in which the two subunits are bonded either by the N-H⋯O hydrogen bond or by van der Waals forces of different type. The differences between the interaction energy (E_int_) and the relative energy (ΔE) values calculated for the 1:1 HNCO⋯SO_2_ complexes are less than 1.55 kJ mol^−1^. Larger differences are predicted for relative Gibbs free energies which is higher by 4–5 kJ mol^−1^ for the two van der Waals species. These predictions were confirmed by the experimental infrared spectra which showed that complexes with hydrogen bonding were more abundant than those with van der Waals interaction. The performed calculations also revealed several stable structures for the HNCO complexes with SO_2_ of the 1:2 stoichiometry. It appeared that the more stable are those containing a N-H⋯O hydrogen bond in addition to the S⋯N and S⋯O van der Waals interactions. These structures give rise to weak bands appearing in the νNH stretching region upon annealing of the matrix.

## Figures and Tables

**Table 1:** Interatomic distances (Å), angles (degree) and electron density parameters of the intermolecular bond critical points (au) of the HNCO complexes with SO_2_ (1:1) computed at the MP2/6-311++G(3df,3pd) level.

Complex	Intermolecular Parameters ^a^			AIM Parameters		
	Interatomic Distances		Angle	BCP	ρ(r)	▽^2^ρ(r)
	H...Y	X⋯Y	X–H...Y			
OS1	2.115	3.121	175.4	H1⋯O6	0.0167	0.0615
OS2	2.115	3.123	178.9	H1⋯O6	0.0172	0.0685
OS3	2.115	3.103	166.0	H1⋯O6	0.0176	0.0694
OS4		3.135		S5⋯N2	0.0110	0.0367
OS5		3.274		S5⋯O4	0.0080	0.0289

^a^ X: N or S; Y: O or N.

**Table 2:** BSSE-corrected interaction energies E_int_, relative energies ΔE, relative Gibbs free energies ΔG (kJ mol^−1^), abundance A (%) and dipole moments μ (Debye) of the HNCO⋯SO_2_ complexes of the 1:1 stoichiometry calculated at MP2, B3LYPD3 and B2PLYPD3 levels.

Complex 1:1	E_int_	ΔE	ΔG	A	μ
	**MP2**				
OS1	−12.68	0.00	0.00	34	4.3
OS2	−12.59	0.11	0.22	32	4.2
OS3	−12.18	0.54	0.88	24	2.8
OS4	−11.13	1.46	4.45	6	1.6
OS5	−11.13	1.53	5.09	4	1.8
	**B3LYPD3**				
OS1	−14.90	0.00	0.00	38	4.5
OS2	−14.77	0.18	0.33	34	4.5
OS3	−14.35	0.60	1.92	18	2.6
OS4	−13.47	1.31	4.46	6	1.7
OS5	−14.48	0.52	5.55	4	1.9
	**B2PLYPD3**				
OS1	−13.77	0.00	0.00	42	4.5
OS2	−13.68	0.08	0.69	31	4.4
OS3	−13.26	0.49	1.97	19	2.8
OS4	−12.30	1.37	5.54	4	1.8
OS5	−12.80	0.95	5.60	4	1.8

**Table 3:** BSSE-corrected interaction energies E_int_, relative energies ΔE, relative Gibbs free energies ΔG (kJ mol^−1^) and dipole moments μ (Debye) of the HNCO⋯SO_2_ complexes of the 1:2 stoichiometry calculated at MP2, B3LYPD3 and B2PLYPD3 levels.

Complex 1:2	E_int_	ΔE	ΔG	μ
	**MP2**			
O2S1	−34.10	0.00	1.71	2.2
O2S2	−33.93	0.17	4.00	2.9
O2S3	−32.22	2.17	8.08	2.3
O2S4	−32.09	2.29	3.28	2.2
O2S5	−20.63	13.38	1.27	2.7
O2S6	−20.50	13.49	0.00	0.7
	**B3LYPD3**			
O2S1	−41.67	0.03	0.00	2.6
O2S2	−41.67	0.00	2.30	2.9
O2S3	−40.17	1.77	5.22	2.2
O2S4	−39.41	2.57	1.05	2.0
O2S5	−26.23	15.15	2.48	2.8
O2S6	−26.15	15.24	1.36	0.8
	**B2PLYPD3**			
O2S1	−38.79	0.00	0.00	2.3
O2S2	−38.62	0.17	2.82	2.9
O2S3	−37.03	2.09	5.91	2.3
O2S4	−36.74	2.39	1.74	2.0
O2S5	−23.93	14.58	2.02	2.8
O2S6	−24.02	14.52	0.59	0.8

**Table 4:** Selected wavenumber shifts calculated for the 1:1 complexes using the MP2, B3LYPD3 and B2PLYPD3 methods with basis set 6-311++G(3df,3pd) compared to the experimental results.

MP2					B3LYPD3					B2PLYPD3					Mode	Exp. ^a^
OS1	OS2	OS3	OS4	OS5	OS1	OS2	OS3	OS4	OS5	OS1	OS2	OS3	OS4	OS5		
−67	−67	−63	−14	−33	−74	−81	−76	−16	−32	−74	−74	−72	−12	−28	νNH	−24.0, −39.5, −49.5, −51.5sh, −66.5
+2	+2	+1	0	−3	0	+1	+1	−3	−2	+1	+1	+1	−1	−3	ν_as_NCO	+1.5, +6.0
−4	−3	−4	−3	−1	−8	−5	−5	−7	−7	−5	−4	−4	−5	−4	ν_asym_SO_2_	−5.5

^a^ The experimental shifts were calculated relative to the corresponding monomer band positions at 3511.5, 1355.0 and 2259.0 cm^−1^, respectively. The underlined value corresponds to the most intense band of the complex in the νNH region.

**Table 5:** Selected wavenumber shifts calculated for the 1:2 complexes using the MP2, B3LYPD3 and B2PLYPD3 methods with basis set 6-311++G(3df,3pd).

MP2						B3LYPD3						B2PLYPD3						Mode
O2S1	O2S2	O2S3	O2S4	O2S5	O2S6	O2S1	O2S2	O2S3	O2S4	O2S5	O2S6	O2S1	O2S2	O2S3	O2S4	O2S5	O2S6	
−118	−123	−74	−82	−19	−15	−143	−145	−68	−91	−21	−18	−132	−137	−74	−88	−17	−14	νNH
−2	−3	−1	1	−1	0	−2	−3	−3	−3	−4	−3	−2	−3	−1	−1	−2	−1	ν_as_NCO
−2	−3	−7	−3	−2	−2	−11	−12	−12	−10	−6	−7	−7	−8	−7	−7	−5	−6	ν_asym_SO_2_
−4	−5	−9	−6	−5	−6	−17	−17	−17	−14	−11	−12	−11	−11	−9	−10	−9	−9	ν_asym_SO_2_

## Data Availability

Not applicable.

## Supplementary Materials

The following are available online. Figure S1: The MP2 optimized structures of the 2:1 complexes of HNCO with SO_2_. Red dots are the RCP’s., Table S1: The MP2 cartesian coordinates of all 1:1, 1:2 and 2:1 optimized species of HNCO with SO_2_, Table S2. Interatomic distances (Å), angles (degree) and electron density parameters of the intermolecular bond critical points BCP (au) and ring critical points RCP(au) of the HNCO complexes with SO_2_ (1:2) computed at the MP2/6-311++G(3df,3pd) level, Table S3: Interatomic distances (Å), angles (degree) and electron density parameters of the intermolecular bond critical points (au) of the HNCO complexes with SO_2_ (2:1) computed at the MP2/6-311++G(3df,3pd) level, Table S4: BSSE-corrected interaction energies E_int_, relative energies ΔE, relative Gibbs free energies ΔG (kJ mol^−1^) and dipole moments μ (Debye) of the HNCO-SO_2_ complexes of the 2:1 stoichiometry calculated at MP2, B3LYPD3 and B2PLYPD3 levels, Table S5: Theoretical infrared wavenumbers ($\bar{\upsilon }$, cm^−1^) and intensities (I, km mol^−1^) for monomers and 1:1 complexes using the MP2, B3LYPD3 and B2PLYPD3 methods with basis set 6-311++G(3df,3pd), Table S6: Theoretical infrared wavenumbers ($\bar{\upsilon }$, cm^−1^) and intensities (I, km mol^−1^) for 1:2 complexes using the MP2, B3LYPD3 and B2PLYPD3 methods with basis set 6-311++G(3df,3pd), Table S7: Theoretical infrared wavenumbers ($\bar{\upsilon }$, cm^−1^) and intensities (I, km mol^−1^) for 2:1 complexes using the MP2, B3LYPD3 and B2PLYPD3 methods with basis set 6-311++G(3df,3pd).

## Sample Availability

Samples of the compounds are not available from the authors.
