# Supplementary material for: Structure and IR Spectroscopic Properties of HNCO Complexes with SO2 Isolated in Solid Argon
Source: Molecules. 2021 Oct 25;26(21):6441. doi: 10.3390/molecules26216441 (PMC8587861; doi:10.3390/molecules26216441)
Supplement: Supplementary file 1 [file molecules-26-06441-s001.zip › molecules-1422206-supplementary.pdf]

Supplementary Materials

Structure and IR spectroscopic properties of HNCO complexes with SO_2 isolated in solid argon

Justyna Krupa, Maria Wierzejewska, Jan Lundell

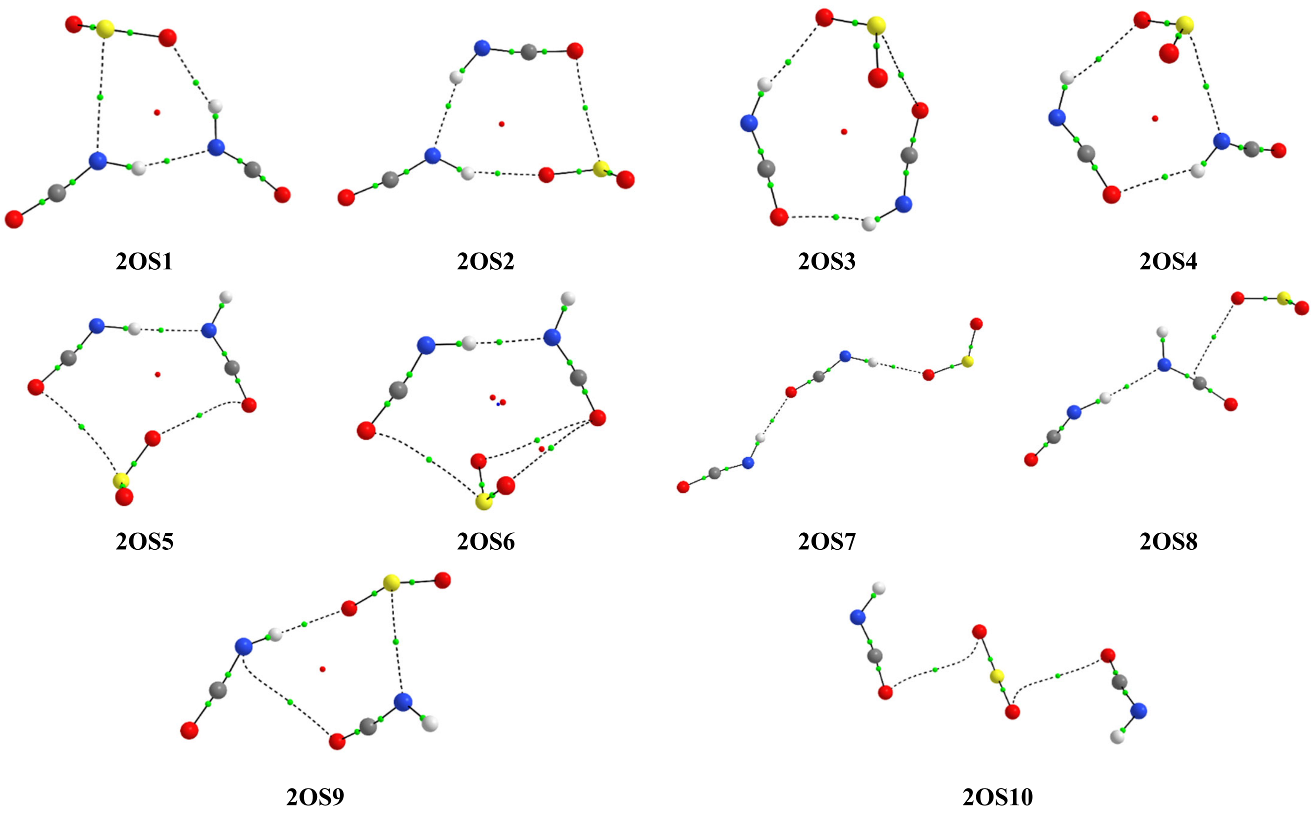

Figure S1. The MP2 optimized structures of the 2:1 complexes of HNCO with SO_2. Red dots are the RCP's.

Table S1. The MP2 cartesian coordinates of all 1:1, 1:2 and 2:1 optimized species of HNCO with SO_2.

Complex 1:1

OS1

0 1

H	1.03577300	-0.89686800	0.00005500
N	2.04278300	-0.94373300	0.00010600
C	2.80132000	0.00683200	0.00000600
O	3.65038300	0.81480600	-0.00007000
S	-2.25101400	-0.11632800	-0.00003600
O	-2.08796700	1.31862600	0.00007500
O	-1.07828500	-0.96802400	-0.00003800

OS2

0 1

H	-1.03270300	0.23319600	0.00040100
N	-1.93398400	0.68486900	0.00055900
C	-3.01319300	0.12452400	0.00003300
O	-4.11722300	-0.26909700	-0.00038800
S	2.29421000	-0.38269400	-0.00002100
O	2.73444700	0.99238700	-0.00031600
O	0.87557300	-0.67970500	0.00018200

OS3

0 1

N	2.04491700	-0.56185800	-0.00000100
C	3.13866800	-0.02848500	0.00000300
H	1.15724200	-0.08434000	-0.00001100
O	4.25169300	0.33661100	0.00000900
O	-3.38809100	0.38821900	0.00000600
O	-0.89262300	0.43666300	-0.00001700
S	-2.12946900	-0.31898000	0.00000100

OS4

0 1

H	1.08364000	-1.47169000	0.46603600
N	1.37385400	-0.69542500	-0.10590600
C	2.44915100	-0.12898000	0.01774500
O	3.42904500	0.50678100	0.01353100
S	-1.64858800	0.09826400	-0.36284400
O	-1.58670300	1.34021800	0.37437000
O	-1.71960700	-1.15433300	0.35889100

OS5

0 1

H	-2.35442200	1.56771700	0.56389500
N	-2.71791000	0.65418700	0.35181300
C	-2.02682500	-0.28516100	0.00825600
O	-1.48974500	-1.27072300	-0.33031500
S	1.50210000	0.05908400	-0.34829400
O	0.61903700	1.19322400	-0.17130800
O	2.05910100	-0.59517600	0.81369600

Complex 1:2

O2S1

0 1

H	-0.45883600	-1.46973900	-0.02945700
N	-1.37378800	-1.14361300	0.25286700
C	-2.40532100	-1.78438700	0.13505200
O	-3.46499300	-2.27602000	0.08676300
O	3.04234500	-0.39538000	1.01120100
S	2.61411900	-0.49124300	-0.36412500
O	1.49241100	-1.34738700	-0.69674100
O	0.46035600	1.59860100	0.05112900
S	-0.93603500	1.85090100	0.34494900
O	-1.82287800	2.22353900	-0.73286300

O2S2

0 1

N	-1.37991100	-1.10174500	-0.57587500
C	-2.27419200	-1.73617100	-0.03999900
H	-0.42659000	-1.42677400	-0.67214200
O	-3.22317400	-2.22260200	0.43903600
S	-0.96568500	1.88855500	-0.34524900
O	-1.44578400	1.98796100	1.01356300
O	0.44443200	1.64925100	-0.57965100
S	2.54507600	-0.49057200	-0.05234000
O	1.63473000	-1.29581200	-0.84361600
O	2.39740300	-0.47026300	1.38375200

O2S3

0 1

H	-1.79206800	1.82455700	-0.56862200
N	-1.07714900	2.50500400	-0.35888900
C	-0.02935600	2.25656600	0.19964800
O	1.02127400	2.16034800	0.71707900
O	-1.30205700	-0.76498300	1.34133000
S	-1.87345000	-1.23135600	0.09859300
O	-2.47582100	-0.26608100	-0.80096000
O	0.89275300	-0.57244900	-1.09122900
S	2.17872300	-0.46260800	-0.42974700
O	2.44183400	-1.28128000	0.73145800

O2S4

0 1

N	0.07432800	2.76843000	0.08980400
C	-1.02617100	2.26207800	0.11249000
H	0.93302500	2.30339300	-0.16550200
O	-2.14325700	1.90150100	0.18129700
S	2.44968200	-0.63403800	-0.33212300
O	2.75892300	-0.87590000	1.05731600
O	2.18162000	0.72089300	-0.77218000
S	-1.96183400	-1.17113500	0.32681000
O	-2.65566300	-1.58800200	-0.86960700
O	-0.52935700	-0.95500500	0.27154300

O2S5

0 1

S	-3.47138000	0.19391900	0.29087400
O	-2.50415000	1.16224000	-0.18252400
O	-3.53944000	-1.10662400	-0.33681100
S	0.42497500	-0.14766700	-0.40087900
O	-0.11241000	-0.71552200	0.81538300
O	1.24877500	1.04039300	-0.31954800
N	4.53191400	0.84249300	0.33555700
C	3.96493000	-0.20233600	0.07802100
H	4.05552100	1.72637100	0.40034000
O	3.55397200	-1.27421800	-0.15866100

O2S6

0 1

H	3.49981300	2.26515100	-0.16143000
N	4.19032500	1.54501900	-0.28962700
C	3.94809300	0.35683400	-0.20000200
O	3.86140200	-0.81096100	-0.14543300
O	1.00421900	0.64291000	0.44835400
S	0.62455800	-0.75433500	0.45838000
O	0.04375100	-1.33254400	-0.73292100
O	-4.53188900	0.60245100	-0.30300700
S	-3.14639300	0.19157400	-0.33296000
O	-2.39889300	0.12100400	0.90577000

Complex 2:1

2OS1

0 1

H	0.47662600	1.06132600	-0.28183300
N	-0.49546100	1.33975000	-0.37731000
C	-0.93689300	2.44328700	-0.10356600
O	-1.49573800	3.44737600	0.11461900
H	1.50505400	-1.11798800	-0.13267900
N	2.03861600	-0.25955500	-0.08363200
C	3.24598700	-0.21651800	0.10137100
O	4.38515900	-0.02625100	0.26962000
O	-0.41783200	-1.92114400	-0.46089800
S	-1.76092200	-1.40861500	-0.26959100
O	-2.27953500	-1.29091500	1.07262500

2OS2

0 1

H	-1.35525000	-0.65805400	-0.29454500
N	-2.16323000	-0.07828700	-0.10714600
C	-3.29256500	-0.51459200	0.05788000
O	-4.41382400	-0.78835700	0.23356000
H	-1.24999100	1.80834800	0.00459400
N	-0.46898200	2.45395800	0.01463900
C	0.70034900	2.14130800	0.05308700
O	1.86420400	1.96969500	0.08603100
O	0.66767100	-1.01030700	-0.73129800
S	2.05353900	-1.08985500	-0.30827400
O	2.34787400	-1.43385700	1.06221700

2OS3

0 1

H	0.10823500	-1.93010900	-0.86204800
N	1.10994000	-2.03704700	-0.79481500
C	1.85416900	-1.45289200	-0.03952500
O	2.69218200	-0.95614400	0.62091700
H	2.42858000	1.22507400	0.51007100
N	1.84868300	1.99950400	0.22384200
C	0.78419600	1.92303100	-0.34891300
O	-0.25361100	1.99757300	-0.89790200
S	-2.12719000	-0.09848100	0.25519000
O	-1.24433300	-0.02414400	1.39725400
O	-1.82452600	-1.05194800	-0.79572200

2OS4

0 1

H	2.14406000	0.77259900	-1.30014400
N	1.99247300	1.71246800	-0.96737700
C	1.22774100	2.00840400	-0.07509900
O	0.52027700	2.43546300	0.76197500
H	-1.17981000	1.09617200	0.73323600
N	-1.58539400	0.25793700	0.33855800
C	-2.74037300	0.17598400	-0.04918800
O	-3.81146800	-0.05208300	-0.45723000
O	1.26653700	-1.11105900	1.30999500
S	0.73595600	-1.76365800	0.13447700
O	1.21049200	-1.34099600	-1.16939900

2OS5

0 1

H	1.20228600	1.83859800	0.16123300
N	0.43970100	2.50002700	0.05714800
C	-0.72857700	2.20954600	-0.08380800
O	-1.88635800	2.06232500	-0.22626000
H	3.54518200	0.96541500	1.01106400
N	2.78974600	0.58196900	0.46481100
C	2.83653600	-0.56965500	0.04515000
O	2.75675700	-1.63658100	-0.41460900
O	-0.25529900	-0.78089300	-0.24763700
S	-1.65266800	-1.15962200	-0.29812700
O	-2.30993300	-1.60277300	0.91050200

2OS6

0 1

H	0.09549300	2.07209700	0.00084900
N	1.09563200	2.23680900	0.00190500
C	1.94262800	1.36794700	0.00082000
O	2.85621900	0.62934300	0.00004900
H	-2.52911400	2.60493100	-0.00332900
N	-1.95856000	1.77453200	-0.00081700
C	-2.45171100	0.65347500	0.00057400
O	-2.78258800	-0.46429200	0.00222400
O	0.18418600	-1.11614600	1.24693200
S	0.50101900	-1.77527400	-0.00212700
O	0.18122200	-1.10897400	-1.24663900

2OS7

0 1

H	1.27797000	0.75608900	-0.04560600
N	0.47376000	1.30890700	0.20841000
C	-0.67881700	1.17423000	-0.12998400
O	-1.83024000	1.17566000	-0.37086500
H	-3.45712500	-0.03573500	-0.05345700
N	-4.16030100	-0.73962600	0.11425300
C	-5.36346900	-0.56781800	0.12062300
O	-6.53533100	-0.55811800	0.15890700
O	4.89895600	0.06181400	0.89383300
S	4.29066400	-0.66712300	-0.19343400
O	2.91512000	-0.38808300	-0.55793400

2OS8

0 1

H	2.72220300	-0.32419400	0.54155100
N	3.69429100	-0.38722000	0.81765400
C	4.66158000	-0.17671300	0.11299200
O	5.68129100	-0.00730100	-0.44158900
H	0.24418500	-1.24318700	0.11629800
N	0.70622400	-0.34660900	0.07121900
C	0.05412500	0.65444900	-0.19722900
O	-0.44557300	1.67950300	-0.44279400
O	-2.39648300	-1.07936700	-0.57829400
S	-3.53878900	-0.27233000	-0.19860400
O	-3.51968600	0.43154700	1.06306800

2OS9

0 1

H	-0.96179800	-1.33075700	0.15184900
N	-1.77744400	-1.34211900	-0.44199500
C	-2.85921700	-0.86431800	-0.15132000
O	-3.94787400	-0.45338400	-0.01962500
H	0.63073600	2.60706900	-1.47458300
N	0.54001100	1.87180400	-0.79307100
C	-0.35020400	1.84916000	0.04554800
O	-1.12738100	1.68106500	0.89993700
O	1.05634900	-0.99232900	0.72325500
S	2.10065900	-0.71965800	-0.24599300
O	3.34879100	-0.15768100	0.21377300

2OS10

0 1

H	-3.37058800	1.48817600	1.33214400
N	-3.88376300	0.69368300	0.98996200
C	-3.40915700	-0.15676800	0.26254000
O	-3.08816800	-1.04353500	-0.43411400
H	3.40104600	-1.44506400	1.37470500
N	3.90037900	-0.65491200	1.00343100
C	3.40954900	0.16831100	0.25590900
O	3.07276100	1.03071900	-0.46355200
O	-0.65228100	1.05826000	-0.03698000
S	-0.00217300	-0.01334800	-0.76049900
O	0.65339400	-1.06672000	-0.01576800

Table S2. Interatomic distances (Å), angles (degree) and electron density parameters of the intermolecular bond critical points BCP (au) and ring critical points RCP(au) of the HNCO complexes with SO_2 (1:2) computed at the MP2/6-311++G(3df,3pd) level.

Complex	Intermolecular parameters[a]			AIM parameters		
	Interatomic distances		Angle	BCP	ρ(r)	$\nabla^2\rho(r)$
	**H···Y**	**X···Y**	**X–H···Y**			
**O2S1**	2.066	3.026	157.7	H1···O6	0.0197	+0.0767
		3.030		S5···O9	0.0108	+0.0402
		3.028		S8···N2	0.0135	+0.0423
			**RCP**	(6 at.)	0.0034	+0.0163
**O2S2**	2.072	3.033	157.6	H1···O6	0.0166	+0.0649
		3.045		S5···O9	0.0093	+0.0338
		3.475		S8···N2	0.0120	+0.0369
			**RCP**	(6 at.)	0.0030	+0.0143
**O2S3**	2.212	3.135	151.4	H1···O6	0.0150	+0.0544
		3.082		S5···O9	0.0105	+0.0356
		3.088		S8···O4	0.0110	+0.0358
		3.472		C3···O7	0.0052	+0.0196
			**RCP1**	HNCOSO	0.0050	+0.0196
			**RCP2**	COSOSO	0.0028	+0.0136
**O2S4**	2.105	3.062	157.5	H1···O6	0.0153	+0.0606
		3.056		S5···O9	0.0090	+0.0324
		3.081		S8···O4	0.0095	+0.0317
			**RCP**	(8 at.)	0.0018	+0.0082
**O2S5**		3.573		C3···O6	0.0070	+0.0288
		3.216		S5···O9	0.0075	+0.0263
**O2S6**		3.293		S5··· O4	0.0081	+0.0289
		3.179		S5···O9	0.0089	+0.0312

[a] X: N, S or C
Y: O or N

Table S3. Interatomic distances (Å), angles (degree) and electron density parameters of the intermolecular bond critical points (au) of the HNCO complexes with SO_2 (2:1) computed at the MP2/6-311++G(3df,3pd) level.

Complex	Intermolecular parameters[a]			AIM parameters		
	Interatomic distances		Angle	BCP	ρ(r)	∇²ρ(r)
	H···Y	X···Y	X–H···Y			
**2OS1**	2.055	3.011	155.9	H1···N6	0.0204	+0.0674
	2.110	2.990	144.2	H5···O10	0.0149	+0.0628
		3.028		S9···N2	0.0119	+0.0369
			**RCP**	(6 at.)	0.0038	+0.0180
**2OS2**	2.099	3.045	154.7	H1···O10	0.0152	+0.0621
		3.091		S9···O8	0.0093	+0.0309
	2.099	3.049	155.3	H5···N2	0.0188	+0.0628
			**RCP**	(8 at.)	0.0025	+0.0111
**2OS3**	2.124	3.095	160.8	H1···O10	0.0157	+0.0575
		3.039		S9···O8	0.0102	+0.0343
	2.200	3.099	147.6	H5···O4	0.0136	+0.0504
			**RCP**	(10 at.)	0.0012	+0.0053
**2OS4**	2.315	3.158	140.5	H1···O10	0.0112	+0.0395
		3.085		S9···N6	0.0107	+0.0332
	2.164	3.058	146.4	H5···O4	0.0143	+0.0553
			**RCP**	(8 at.)	0.0021	+0.0101
**2OS5**	2.047	3.061	176.3	H1···N6	0.0211	+0.0653
		3.136		O8···O10	0.0055	+0.0234
		3.231		S9···O4	0.0075	+0.0264
			**RCP**	(9 at.)	0.0028	+0.0122
**2OS6**	2.076	3.089	178.9	H1···N6	0.0197	+0.0627
		3.283		O8···O10	0.0048	+0.0184
		3.280		O8···O11	0.0048	+0.0184
		3.366		S9···O4	0.0058	+0.0229
			**RCP1**	(9 at.)	0.0017	+0.0074
			**RCP2**	(9 at.)	0.0017	+0.0075
			**RCP3**	(4 at.)	0.0044	+0.0198
**2OS7**	2.053	3.055	171.7	H1···O8	0.0165	+0.0672
	2.062	3.070	179.5	H5···O10	0.0166	+0.0643
**2OS8**	2.070	3.080	175.0	H1···N6	0.0200	+0.0635
		3.026		C7···O10	0.0071	+0.0273
**2OS9**	2.125	3.084	158.1	H1···O10	0.0151	+0.0590
		3.074		S9···N6	0.0113	+0.0345
		3.371		O8···N2	0.0053	+0.0180
			**RCP**	(7 at.)	0.0026	+0.0124
**2OS10**		3.242		O4···O10	0.0071	+0.0262
		3.233		O11···O8	0.0072	+0.0262

[a] X: N, O, S or C
Y: O or N

Table S4. BSSE corrected interaction energies E_{int}, relative energies ΔE, relative Gibbs free energies ΔG (kJ mol^{-1}) and dipole moments μ (Debye) of the HNCO-SO_2 complexes of the 2:1 stoichiometry calculated at MP2, B3LYPD3 and B2PLYPD3 levels.

Complex 2:1	E_{int}	ΔE	ΔG	μ
		**MP2**		
**2OS1**	-40.96	0.00	8.4	2.1
**2OS2**	-39.12	2.24	11.0	2.5
**2OS3**	-37.24	4.34	14.3	2.2
**2OS4**	-35.82	5.24	14.4	1.6
**2OS5**	-32.09	9.42	12.8	2.9
**2OS6**	-31.55	9.61	12.2	3.1
**2OS7**	-29.08	12.24	0.0	6.4
**2OS8**	-28.33	13.04	6.9	5.5
**2OS9**	-27.78	12.81	16.3	3.3
**2OS10**	-22.64	17.92	12.1	0.2
		**B3LYPD3**		
**2OS1**	-47.20	0.00	5.2	2.0
**2OS2**	-45.56	2.17	6.9	2.6
**2OS3**	-45.02	2.77	10.6	2.2
**2OS4**	-43.68	3.69	8.5	1.4
**2OS5**	-36.53	11.17	9.8	2.7
**2OS6**	-36.40	10.82	9.1	3.1
**2OS7**	-33.39	14.01	0.0	6.7
**2OS8**	-32.72	14.77	6.4	5.7
**2OS9**	-32.89	13.80	13.4	3.6
**2OS10**	-29.20	17.54	10.9	0.2
		**B2PLYPD3**		
**2OS1**	-44.10	0.00	6.3	2.0
**2OS2**	-42.68	1.95	8.8	2.5
**2OS3**	-41.17	3.72	11.7	2.1
**2OS4**	-39.46	4.87	9.9	1.4
**2OS5**	-34.06	10.60	11.1	2.8
**2OS6**	-33.64	10.61	10.8	3.0
**2OS7**	-31.34	13.04	0.0	6.7
**2OS8**	-30.08	14.21	6.1	5.5
**2OS9**	-30.12	13.61	14.7	3.6
**2OS10**	-25.90	17.75	10.9	0.4

Table S5. Theoretical infrared wavenumbers (ῡ, cm^{-1}) and intensities (I, km mol^{-1}) for monomers and 1:1 complexes using the MP2, B3LYPD3 and B2PLYPD3 methods with basis set 6-311++G(3df,3pd).

Mode	OS1		OS2		OS3		OS4		OS5		HNCO		SO_2	
	ῡ	I	ῡ	I	ῡ	I	ῡ	I	ῡ	I	ῡ	I	ῡ	I
MP2														
δSO_2	505	23	504	32	508	24	505	24	506	25			503	25
δNCO	606	53	606	51	604	85	563	112	585	78	571	94		
γNCO	648	0	648	0	646	0	634	1	637	1	636	1		
δHNC	797	221	797	259	795	318	780	261	816	259	785	215		
$\nu_{sym}SO_2$	1130	32	1130	29	1130	21	1131	19	1133	19			1128	19
$\nu_{sym}NCO$	1308	4	1308	5	1308	6	1308	2	1304	1	1306	0		
$\nu_{asym}SO_2$	1346	166	1347	184	1347	199	1348	151	1349	149			1350	154
$\nu_{asym}NCO$	2338	697	2339	745	2338	744	2337	616	2334	749	2337	675		
νNH	3663	603	3662	663	3666	635	3715	176	3697	158	3729	175		
B3LYPD3														
δSO_2	524	34	521	43	526	30	522	27	522	29			519	29
δNCO	613	40	615	38	616	78	568	98	596	62	576	83		
γNCO	649	1	650	1	648	1	637	3	641	2	639	3		
δHNC	823	188	817	239	815	337	803	263	830	237	798	205		
$\nu_{sym}SO_2$	1177	47	1178	48	1177	33	1178	29	1178	31			1178	31
$\nu_{sym}NCO$	1338	3	1339	10	1338	14	1337	2	1335	0	1336	0		
$\nu_{asym}SO_2$	1370	201	1372	250	1372	271	1371	215	1370	209			1377	221
$\nu_{asym}NCO$	2330	753	2331	837	2332	835	2328	676	2328	858	2330	757		
νNH	3606	512	3600	698	3604	647	3664	159	3649	147	3681	159		
B2PLYPD3														
δSO_2	513	26	512	36	517	26	513	25	514	27			511	27
δNCO	611	41	612	39	611	70	569	93	588	55	575	79		
γNCO	647	0	647	0	645	0	633	2	635	2	635	2		
δHNC	821	207	821	250	819	327	799	267	840	235	803	213		
$\nu_{sym}SO_2$	1140	40	1140	37	1139	26	1140	23	1141	24			1138	23
$\nu_{sym}NCO$	1317	5	1317	9	1317	13	1317	3	1313	1	1315	0		
$\nu_{asym}SO_2$	1343	192	1344	216	1344	232	1343	181	1344	178			1348	185
$\nu_{asym}NCO$	2320	713	2320	768	2320	767	2318	636	2316	772	2319	696		
νNH	3628	601	3627	681	3630	651	3690	163	3674	145	3702	159		

Table S6. Theoretical infrared wavenumbers ($\bar{\upsilon}$, cm^{-1}) and intensities (I, km mol^{-1}) for 1:2 complexes using the MP2, B3LYPD3 and B2PLYPD3 methods with basis set 6-311++G(3df,3pd).

Mode	O2S1		O2S2		O2S3		O2S4		O2S5		O2S6	
	$\bar{\upsilon}$	I	$\bar{\upsilon}$	I	$\bar{\upsilon}$	I	$\bar{\upsilon}$	I	$\bar{\upsilon}$	I	$\bar{\upsilon}$	I
MP2												
δSO_2	507	4	507	24	507	18	506	12	504	15	506	15
δSO_2	507	54	510	27	511	43	508	36	508	35	509	32
δNCO	617	50	615	46	597	58	601	52	562	110	562	112
γNCO	645	1	647	2	641	0	642	0	635	1	635	1
δHNC	843	366	844	309	802	263	791	267	786	271	781	268
$\nu_{sym}SO_2$	1136	28	1134	32	1131	14	1133	19	1128	22	1127	16
$\nu_{sym}SO_2$	1136	24	1135	21	1131	34	1135	29	1134	19	1134	20
$\nu_{sym}NCO$	1306	1	1305	2	1309	3	1310	7	1308	2	1308	2
$\nu_{asym}SO_2$	1346	63	1345	137	1341	11	1345	151	1345	60	1344	231
$\nu_{asym}SO_2$	1348	293	1347	188	1343	265	1347	196	1348	240	1348	104
$\nu_{asym}NCO$	2335	815	2334	781	2336	592	2338	657	2336	602	2337	606
νNH	3611	581	3606	536	3655	318	3647	492	3711	169	3714	171
B3LYPD3												
δSO_2	523	8	522	29	524	20	523	14	521	17	523	12
δSO_2	525	66	531	26	529	52	526	38	525	40	526	42
δNCO	631	26	625	28	607	48	602	47	568	96	568	97
γNCO	649	4	654	3	643	2	644	1	638	3	638	3
δHNC	874	324	873	241	820	264	813	246	812	275	806	272
$\nu_{sym}SO_2$	1175	22	1175	22	1175	29	1177	33	1178	24	1176	15
$\nu_{sym}SO_2$	1179	63	1178	68	1178	49	1179	48	1180	41	1180	38
$\nu_{sym}NCO$	1337	4	1336	5	1339	3	1339	7	1336	2	1336	2
$\nu_{asym}SO_2$	1361	199	1360	167	1360	13	1363	155	1366	134	1366	283
$\nu_{asym}SO_2$	1366	288	1366	266	1366	376	1368	318	1372	294	1370	197
$\nu_{asym}NCO$	2329	939	2327	901	2327	672	2327	731	2327	662	2327	666
νNH	3537	616	3536	537	3612	304	3590	451	3659	153	3663	155
B2PLYPD3												
δSO_2	514	5	514	26	516	21	513	11	512	17	514	12
δSO_2	516	61	520	29	520	45	516	39	517	36	517	38
δNCO	626	35	622	33	603	46	603	42	568	91	568	92
γNCO	644	2	648	3	639	1	640	0	634	2	634	2
δHNC	874	360	874	295	827	270	818	262	808	280	802	277
$\nu_{sym}SO_2$	1142	10	1141	13	1138	21	1141	24	1138	26	1137	16
$\nu_{sym}SO_2$	1144	56	1142	57	1140	42	1143	39	1142	25	1143	26
$\nu_{sym}NCO$	1316	3	1315	4	1317	3	1318	8	1316	2	1316	3
$\nu_{asym}SO_2$	1337	135	1337	153	1334	12	1337	154	1339	71	1339	264
$\nu_{asym}SO_2$	1341	287	1340	229	1338	321	1341	257	1342	289	1341	142
$\nu_{asym}NCO$	2317	860	2316	832	2316	626	2317	680	2317	623	2317	627
νNH	3570	586	3565	540	3622	325	3614	459	3685	156	3688	158

Table S7. Theoretical infrared wavenumbers (ῡ, cm^{-1}) and intensities (I, km mol^{-1}) for 2:1 complexes using the MP2, B3LYPD3 and B2PLYPD3 methods with basis set 6-311++G(3df,3pd).

Mode	2OS1		2OS2		Mode	2OS3		2OS4		2OS7		2OS8		2OS9		2OS10		Mode	2OS5		2OS6	
	ῡ	I	ῡ	I		ῡ	I	ῡ	I	ῡ	I	ῡ	I	ῡ	I	ῡ	I		ῡ	I	ῡ	I
MP2																						
δSO_2	508	36	508	30	δSO_2	512	34	511	36	504	35	505	33	512	22	507	22	δSO_2	506	25	507	34
δNCO	594	164	592	24	δNCO	599	58	586	78	612	16	581	100	578	38	563	149	δNCO	569	56	567	45
γNCO	638	1	636	31	δNCO	609	56	612	76	613	105	623	28	603	73	564	79	γNCO	629	17	630	1
γNCO	644	5	643	5	γNCO	640	0	636	1	647	0	632	1	632	1	635	1	δNCO	632	7	632	22
δNCO	660	4	649	2	γNCO	648	0	645	7	650	0	668	6	648	1	635	0	γNCO	657	3	646	0
δHNC	825	559	809	193	δHNC	777	449	786	358	757	403	814	318	801	136	774	386	δHNC	821	255	823	177
δHNC	915	235	886	494	δHNC	797	56	854	241	803	234	882	260	834	310	781	147	δHNC	889	276	881	309
$\nu_{sym}SO_2$	1137	27	1135	29	$\nu_{sym}SO_2$	1131	28	1132	24	1131	32	1129	23	1133	18	1134	19	$\nu_{sym}SO_2$	1130	19	1129	21
$\nu_{sym}NCO$	1305	2	1305	1	$\nu_{sym}NCO$	1309	1	1304	0	1309	4	1301	1	1302	0	1308	3	$\nu_{sym}NCO$	1299	3	1301	1
$\nu_{sym}NCO$	1306	1	1309	10	$\nu_{sym}NCO$	1310	7	1309	3	1317	28	1308	7	1307	4	1309	1	$\nu_{sym}NCO$	1308	10	1308	6
$\nu_{asym}SO_2$	1347	173	1344	175	$\nu_{asym}SO_2$	1340	132	1341	128	1346	189	1345	145	1345	183	1346	149	$\nu_{asym}SO_2$	1346	164	1344	129
$\nu_{asym}NCO$	2329	1127	2330	1479	$\nu_{asym}NCO$	2323	97	2332	1032	2337	1372	2327	905	2331	283	2337	773	$\nu_{asym}NCO$	2327	576	2327	466
$\nu_{asym}NCO$	2341	550	2343	120	$\nu_{asym}NCO$	2352	1020	2337	379	2351	339	2341	521	2337	954	2338	457	$\nu_{asym}NCO$	2343	719	2344	786
νNH	3525	764	3567	560	νNH	3634	262	3619	355	3642	1452	3569	981	3650	512	3715	153	νNH	3534	872	3557	769
νNH	3613	540	3610	751	νNH	3660	502	3671	296	3658	229	3666	160	3702	178	3716	198	νNH	3684	167	3690	176
B3LYPD3																						
δSO_2	524	53	526	41	δSO_2	528	42	529	41	522	49	523	37	528	28	524	24	δSO_2	522	30	525	41
δNCO	612	125	602	17	δNCO	606	31	593	65	620	6	599	88	588	32	568	125	δNCO	579	49	576	37
γNCO	640	2	643	8	δNCO	612	70	624	58	623	85	629	26	615	62	569	76	γNCO	635	7	633	3
γNCO	647	3	648	3	γNCO	642	2	637	5	649	1	635	4	635	4	638	2	δNCO	640	17	642	19
δNCO	676	7	654	18	γNCO	649	2	648	10	654	0	673	6	652	1	638	4	γNCO	658	3	648	0
δHNC	849	539	831	221	δHNC	797	431	811	370	777	365	833	270	819	125	796	394	δHNC	843	237	843	164
δHNC	945	201	915	433	δHNC	819	73	874	197	825	234	909	291	848	301	804	144	δHNC	908	272	901	298
$\nu_{sym}SO_2$	1176	47	1178	45	$\nu_{sym}SO_2$	1176	38	1176	42	1178	55	1178	36	1175	29	1178	27	$\nu_{sym}SO_2$	1179	30	1180	33
$\nu_{sym}NCO$	1337	5	1337	7	$\nu_{sym}NCO$	1340	3	1337	1	1339	7	1331	1	1334	0	1337	3	$\nu_{sym}NCO$	1330	2	1333	1
$\nu_{sym}NCO$	1338	2	1338	8	$\nu_{sym}NCO$	1340	6	1338	4	1344	42	1338	11	1338	12	1337	1	$\nu_{sym}NCO$	1338	12	1338	8
$\nu_{asym}SO_2$	1361	230	1365	233	$\nu_{asym}SO_2$	1358	180	1359	185	1371	249	1371	205	1364	249	1364	211	$\nu_{asym}SO_2$	1371	229	1369	186
$\nu_{asym}NCO$	2321	1299	2320	1590	$\nu_{asym}NCO$	2313	141	2325	1190	2328	1693	2319	1141	2326	156	2328	819	$\nu_{asym}NCO$	2318	657	2319	530
$\nu_{asym}NCO$	2337	653	2338	211	$\nu_{asym}NCO$	2345	1121	2332	449	2342	201	2335	484	2333	1276	2329	531	$\nu_{asym}NCO$	2338	816	2340	893
νNH	3437	806	3492	550	νNH	3593	127	3564	392	3571	1457	3497	1022	3594	530	3665	86	νNH	3463	893	3490	783
νNH	3543	572	3542	823	νNH	3609	588	3628	271	3590	300	3620	149	3661	167	3665	232	νNH	3640	151	3648	162

B2PLYPD3																						
δSO_2	515	42	516	36	δSO_2	521	36	520	34	512	40	513	33	520	25	515	23	δSO_2	514	27	516	37
δNCO	605	133	598	15	δNCO	604	41	588	63	616	8	590	88	586	32	568	123	δNCO	579	48	574	36
γNCO	637	1	638	14	δNCO	613	51	623	65	618	81	624	26	611	58	569	66	γNCO	630	11	629	2
γNCO	645	3	644	11	γNCO	639	1	634	2	646	0	631	2	631	3	633	1	δNCO	633	12	635	19
δNCO	663	5	647	8	γNCO	646	1	643	7	650	0	668	6	648	1	633	2	γNCO	657	3	645	0
δHNC	851	572	835	211	δHNC	801	450	811	407	782	381	836	293	826	144	794	422	δHNC	845	237	847	165
δHNC	945	214	914	455	δHNC	824	60	883	224	828	237	903	277	858	298	801	128	δHNC	909	276	905	305
$\nu_{sym}SO_2$	1143	35	1142	36	$\nu_{sym}SO_2$	1140	33	1141	32	1140	42	1139	28	1140	23	1142	22	$\nu_{sym}SO_2$	1141	24	1140	25
$\nu_{sym}NCO$	1315	4	1316	5	$\nu_{sym}NCO$	1318	3	1315	1	1318	6	1310	1	1312	0	1317	5	$\nu_{sym}NCO$	1309	2	1311	1
$\nu_{sym}NCO$	1317	1	1317	9	$\nu_{sym}NCO$	1318	8	1317	4	1324	44	1317	9	1317	12	1317	1	$\nu_{sym}NCO$	1317	11	1317	7
$\nu_{asym}SO_2$	1338	203	1338	202	$\nu_{asym}SO_2$	1333	158	1334	160	1343	213	1342	175	1338	213	1338	178	$\nu_{asym}SO_2$	1342	196	1341	157
$\nu_{asym}NCO$	2310	1196	2309	1487	$\nu_{asym}NCO$	2303	121	2314	1205	2318	1490	2309	997	2313	212	2317	817	$\nu_{asym}NCO$	2308	591	2308	475
$\nu_{asym}NCO$	2324	575	2325	169	$\nu_{asym}NCO$	2334	1060	2319	298	2332	263	2323	490	2320	1099	2319	455	$\nu_{asym}NCO$	2326	760	2327	827
νNH	3483	756	3529	522	νNH	3599	245	3580	408	3603	1477	3536	969	3616	536	3690	93	νNH	3498	864	3523	759
νNH	3574	567	3572	793	νNH	3625	537	3641	293	3620	241	3644	149	3677	165	3690	231	νNH	3660	153	3666	161
